# Locus of Control, Learning Styles, and Academic Achievement of Saudi Pre-professional Medical Students: A Cross-sectional Study

**DOI:** 10.1007/s40596-024-02056-9

**Published:** 2024-09-30

**Authors:** Suha M. Althubaiti, Nouf S. Alharbi, Alaa Althubaiti, Amal Alzahrani, Sajida Agha

**Affiliations:** 1 College of Science and Health Professions, King Saud Bin Abdulaziz University for Health Sciences, Riyadh, Saudi Arabia; 2 King Abdullah International Medical Research Center, Riyadh, Saudi Arabia; 3 College of Medicine, King Saud Bin Abdulaziz University for Health Sciences, Riyadh, Saudi Arabia; 4 College of Medicine, King Saud Bin Abdulaziz University for Health Sciences, Jeddah, Saudi Arabia

**Keywords:** Learning style, Locus of control, Academic achievement

## Abstract

**Objective:**

The objective of this study was to describe medical students’ learning styles and locus of control (LOC) and to determine their association with academic achievement.

**Methods:**

A cross-sectional study was performed at a health science university in Saudi Arabia. A sample of 158 medical students completed Kolb’s learning style and locus of control scales. The authors measured academic achievement by grade point average (GPA) and collected demographic characteristics.

**Results:**

Most students’ learning styles were classified as convergent (51.3%), whereas the rest adopted divergent (23.4%), accommodative (18.4%), and assimilative (7%) styles. Analysis revealed that students with a lower LOC tend to have higher GPAs than those with a higher LOC. However, no association was found between Kolb’s learning styles and LOC or GPA.

**Conclusions:**

The findings of this study provide educators with essential insights into factors that enhance students’ academic achievements. We conclude that locus of control plays a crucial role in academic success. Medical educators should consider diverse student learning styles to adopt appropriate teaching methods that meet students’ needs, ultimately improving educational practices and outcomes in medical education.

The academic achievement of students, indicated by their grade point average (GPA), is a critical element in educational assessment. This achievement is influenced by various factors, including the student’s preferred learning style, which is shaped by their locus of control (LOC) beliefs. Learning styles pertain to how students prefer to process, comprehend, and retain information, suggesting that certain instructional methods are more effective for learning [1].

Research has explored the relationship between LOC, learning styles, and academic success, aiming to enhance understanding and optimize student information delivery [1, 2]. Studies have also investigated how culture and sex impact LOC, with findings indicating that females tend to exhibit more external LOC beliefs than males that could be due to cultural influences or conformity [3].

Furthermore, individuals possess unique attributes and may explore various learning styles to cater to their cognitive differences. The Kolb experiential model outlines a cyclic problem-solving approach to learning, encompassing concrete experience, reflective observation, abstract conceptualization, and active experimentation [1]. This model helps determine a student’s dominant learning style based on their approach to resolving tensions between experiences and concepts, as well as between action and reflection. Understanding these dynamics can aid in tailoring educational practices to accommodate diverse learning preferences and optimize academic outcomes.

Prior studies have demonstrated a significant correlation between LOC and academic performance, indicating that students with an internal LOC tend to achieve higher academic success compared to those with an external LOC [4]. This suggests that students with internal LOC who believe their efforts directly influence their outcomes are generally more successful academically. However, there is a notable gap in the research regarding the impact of these factors on medical students in Saudi Arabia. To address this gap, we have designed a study focused on examining the learning styles and LOC of medical students in Saudi Arabia. Medical education in Saudi Arabia has undergone significant transformations to align with international standards, emphasizing innovative teaching strategies and critical thinking skills. This evolving landscape highlights the relevance of this research in addressing current educational challenges. The primary aims of this study are to describe the learning styles and LOC among these students, and to determine the association between these variables and their academic achievement.

## Methods

The study was conducted at a medical school at King Saud Bin Abdulaziz University for Health Sciences, Riyadh, Saudi Arabia. A convenient non-probability sampling technique was applied. The sample included medical students who completed the fourth semester of the pre-professional medical program for the academic year 2021–2022. After the Institutional Review Board approval (IRB/1171/22) from King Abdullah International Medical Research Center (KAIMRC), was obtained, a list of all students was requested. Participation in the study was limited to those who consented. The total pool of students consisted of 170 male and 103 female medical students. In the end, 158 students took part in the study, resulting in an overall response rate of 57.9%.

Data was collected online using validated questionnaires and demographic forms, with a pilot test ensuring clarity and reliability. To minimize potential reporting bias, students’ grades were directly obtained from the university’s assessment unit for an accurate evaluation of academic performance. The questionnaire’s first part included demographic characteristics such as sex, type of previous school attended (private or public), and age, which were previously found to be associated with learning styles [5]. In our sample, all students are from the same academic level, so the academic level was not collected.

To gather the necessary data for this study, two main tools were employed: the Trice Academic Locus of Control scale for college students and the Kolb Learning Style Inventory. The Trice Academic LOC scale is a 28-item questionnaire that measures an individual’s internal versus external control of reinforcement with various aspects of university students’ lives, including GPA, final exam grades, and attendance [6]. Students respond to statements using a true or false scale, and reliability analysis yielded an acceptable internal consistency. Locus of control scores range from 0 to 28, with high scores indicating more external orientation.

The Kolb Learning Style Inventory [7] comprises a 10-item questionnaire in which students rank four words according to their learning style preferences. These preferences reveal their dominant learning ability, categorized into Diverging, Assimilating, Converging, and Accommodating styles. Students assign a rank from 4 (best) to 1 (least) for each set of four words in the items. This ranking system helps determine their dominant learning style.

The study utilized JMP 14 software for analyses (SAS Institute Inc., Cary, NC, USA). Descriptive statistics captured categorical variables as frequencies and percentages. The Shapiro–Wilk test assessed normal distribution. Non-normally distributed continuous variables were presented as median and quartiles. Spearman’s correlation explored LOC and learning style associations. Chi-squared or Fisher’s exact test compared GPA and demographics across learning styles. Kruskal–Wallis tested LOC among GPA subgroups. Mann–Whitney *U* test assessed pairwise differences. Data cleaning eliminated incomplete rows. Multinomial logistic regression identified variables linked to GPA. Significance was set at *p* < 0.05.

## Results

Table 1 presents the associations between demographic characteristics, learning styles, and GPA for medical students. The majority of students (51.3%) exhibit a convergent learning style, followed by those with divergent (23.4%), accommodative (18.4%), and assimilative (7%) styles. An analysis of GPA distribution across these learning styles revealed no statistically significant differences in GPA categories among the different learning styles (chi-squared = 3.098, *p*-value = 0.79). Bivariate correlations between the classifications of learning styles are statistically significant. A moderate negative correlation was found between concrete experience and active experimentations (Spearman rank correlation, Rs =  − 0.69, *p*-value < 0.0001). The highest score was for active experimentation, with a median of 39, and the lowest was for reflective observation and concrete experience.

**Table 1:** Baseline characteristics and learning styles of medical students

Variable	Categories	All *n*(%)	GPA			Test statistics value	*p*-value
			4.75–5 (*n* = 66)	4.5 to less than 4.75 (*n* = 44)	3.75 to less than 4.5 (*n* = 48)		
Sex	Female	90(57)	58(64.4)	26(28.9)	6(6.7)	64.52	< 0.0001*
	Male	68(43)	8(11.8)	18(26.5)	42(61.8)		
Age (years)	18–21	139(88)	56(40.3)	37(26.6)	46(33.1)	4.78	0.11^†^
	22–25	19(12)	10(52.6)	7(36.8)	2(10.5)		
Schools	Public	91(57.6)	30(33.0)	27(29.7)	34(37.4)	7.68	0.021*
	Private	67(42.4)	36(53.7)	17(25.4)	14(20.9)		
Learning styles	Convergent	81(51.3)	30(37)	26(32.1)	25(30.9)	3.098	0.79^†^
	Divergent	37(23.4)	17(45.9)	8(21.6)	12(32.4)		
	Accommodative	29(18.4)	15(51.9)	7(24.1)	7(24.1)		
	Assimilative	11(7)	4(36.4)	3(27.3)	4(36.4)		

Total number of students 158

^*^Pearson Chi-square test

^†^Fisher’s exact test

The locus of control is not significantly correlated with learning styles (*p*-value > 0.05). A comparison of LOC scores among GPA groups is presented in Table 2. Results revealed a statistically significant association between these variables (*p*-value = 0.014). From the results, it can be concluded that the median LOC was higher for students with a GPA of 3.75 to < 4.5 (median LOC 14) than for students with a GPA of 4.75–5 (median LOC 11) (moderate effect size: 0.22; *p*-value = 0.006). A moderate effect size suggests that the difference in LOC between these GPA groups might have some practical relevance in understanding academic performance variability. The median LOC was higher for students with GPAs 4.5 to < 4.75 than for students with GPAs 4.75–5 (small effect size: 0.18, *p*-value = 0.021). A small effect size indicates a potentially subtle difference in LOC among these GPA groups. In addition, a Mann–Whitney *U* test showed a statistically significant association between sex and LOC (moderate effect size: 0.30, *p*-value < 0.001), where female students had lower LOC than male students (median score [Q1–Q3]: 11 [9–13.25] versus 14 [12–17]). No statistically significant association was found between the type of school attended and LOC (chi-squared test = 0.034; *p*-value = 0.85).

**Table 2:** Comparison of total academic locus of control and academic achievement

Variable	Group		Locus of control Median (Q1–Q3)	Test value	*p*-value*	Comparison group	Effect size	Post hoc *p*-value**
GPA	(1)	4.75–5	11(9–14.25)	9.126	0.014	1 vs 2	0.18	0.021
	(2)	4.5 to less than 4.75	13(11–16.75)			1 vs 3	0.22	0.006
	(3)	3.75 to less than 4.5	14(11–15.75)			2 vs 3	0.03	0.71

Total number of students 158

*GPA*, grade point average

^*^Kruskal–Wallis test

^**^Post hoc (Mann–Whitney *U*) test

To further explore the significant association between GPA and LOC, we used a multinomial logistic regression analysis with GPA as the dependent variable and LOC, sex, and type of school as independent variables. The results revealed that female students are related to increased odds of a GPA of 4.75–5 or a GPA of 4.5 to less than 4.75 versus a GPA of 3.75 to less than 4.5 compared to male students (OR = 4.3, 95%CI 2.9–5.6, *p*-value < 0.0001; OR = 2.7, 95%CI 1.5–3.8, *p*-value < 0.0001, respectively). Locus of control and last school attended were not independently and significantly associated with GPA.

## Discussion

Our study on the preferred learning styles and LOC among medical students has yielded significant insights. There is a notable gap in the research regarding the impact of these factors on medical students in Saudi Arabia This research provides an in-depth analysis of LOC, the learning styles adopted by students, and their impact on academic achievements, thereby offering a better understanding of the students’ potential. A statistically significant association was found between the LOC and GPA, aligning with the findings of other researchers [4, 8]. Students with a lower LOC score, indicating an internal LOC, exhibit traits such as higher endurance, motivation, and independence compared to those with a higher LOC score, which indicates an external LOC [8].

Our findings revealed that female students exhibited a lower LOC and achieved higher GPAs than their male counterparts. Several factors may contribute to this lower LOC among female students. For instance, societal expectations and cultural norms often emphasize personal accountability and empowerment for women, which fosters a greater sense of control over their lives and supports their belief in their ability to influence their circumstances [9]. In Saudi Arabia, the growing emphasis on women’s empowerment and belief in their abilities may influence the relationship between LOC and academic achievement [4, 8]. In contrast to our findings, female accounting students demonstrated higher levels of LOC and better academic performance compared to male students [3]. However, similar to our results, total LOC was not significantly associated with higher performance for either gender. This suggests that gender may have a stronger correlation with academic performance than LOC does. This highlights gender as a significant factor in LOC that is linked to increased academic success. Understanding these differences can help educators and mentors provide tailored support to students based on their individual needs and beliefs about control.

Research on the association between learning styles and the academic performance of students has generated conflicting results and highlighted several factors. On one hand, some studies, including ours, have found no significant relationship between learning styles and GPA [10], while others have reported correlations. For instance, medical students with diverging learning styles (empathetic, imaginative) achieve higher GPAs through self-reflection, unlike theory-focused assimilators [11]. Research on medical students in Saudi Arabia and Japan revealed a connection between sex, type of school, and GPA [12, 13]. Specifically, female medical students exhibit greater motivation than males [14]. Furthermore, internal motivation is crucial for academic success in the preclinical years, shedding light on academic achievement factors such as motivation levels [15]. Additionally, the learning environment and independent learning were found to influence GPA [16]. These findings imply that the relationship between learning styles and academic achievement is complicated and can be influenced by various factors such as sex, internal motivation, and learning environment.

Cultural factors in Saudi Arabia have a significant influence on learning styles and LOC among students. Learning styles are affected by sociocultural factors including religion, Arabic as the first language, and teaching methods [17]. Studies show that an internal LOC is associated with better self-regulated learning skills and learning retention in flipped learning environments [18]. Cross-cultural comparisons reveal differences in learning style preferences among Saudi students and those from other countries [19]. Future research should explore these dynamics further, suggesting avenues for comparative studies across different cultural settings to understand better how cultural variations influence learning styles and LOC.

Our study revealed that medical students predominantly favor a convergent learning style over other Kolb styles. According to the learning styles hypothesis, learners can only reach their full potential when teaching methods align with their preferred learning styles. Therefore, identifying the most effective instructional strategies based on students’ learning styles allows educators to optimize their teaching and training approaches to achieve educational objectives. These findings hold significant implications for medical education in Saudi Arabia, underscoring the importance for educators to consider these preferences in their curriculum design and pedagogical techniques. Educators can enhance instructional methods by aligning teaching strategies with students’ preferred learning styles, fostering an internal locus of control through goal setting and self-assessment, and differentiating instruction based on individual needs. Furthermore, future research may investigate longitudinal studies to track changes in learning styles and locus of control over time, helping to provide a more comprehensive understanding of how these variables evolve throughout medical education and potentially refine teaching strategies accordingly.

This study had several limitations. Firstly, it was conducted at a single institution, which may limit the generalizability of the findings to other settings or populations. Additionally, the study relied on self-reported data, which can be susceptible to bias and inaccuracies [20]. Despite these limitations, the study’s findings are invaluable, as they can help identify students at risk of poor academic achievement. This, in turn, can inform the development of targeted monitoring strategies and interventions to support these students in enhancing their academic performance.

## Data Availability

The data that support the findings of this study are available from the corresponding author upon reasonable request.
